# Perspectives on extended-release naltrexone induction among patients living with HIV and opioid use disorder: a qualitative analysis

**DOI:** 10.1186/s13722-021-00277-z

**Published:** 2021-11-10

**Authors:** Kim A. Hoffman, Robin Baker, Laura C. Fanucchi, Paula J. Lum, Lynn E. Kunkel, Javier Ponce Terashima, Dennis McCarty, Petra Jacobs, P. Todd Korthuis

**Affiliations:** 1 Oregon Health and Science University-Portland State University School of Public Health, Portland, OR USA; 2 College of Medicine, University of Kentucky, Lexington, KY USA; 3 Department of Medicine, Division of HIV, ID & Global Medicine, University of California San Francisco, San Francisco, CA USA; 4 Department of Medicine, Section of Addiction Medicine, Oregon Health & Science University, Portland, OR USA; 5 Yale University, New Haven, CT USA; 6 National Institute on Drug Abuse, Center for the Clinical Trials Network, North Bethesda, MD USA

**Keywords:** Extended-release naltrexone, Opioid withdrawal, Induction, Opioid use disorder, HIV

## Abstract

**Background:**

The CHOICES study randomized participants with HIV and opioid use disorder (OUD) to HIV clinic-based extended-release naltrexone (XR-NTX), which requires complete cessation of opioid use, versus treatment-as-usual (i.e., buprenorphine, methadone). Study participants randomized to XR-NTX were interviewed to assess their experiences with successful and unsuccessful XR-NTX induction.

**Methods:**

Semi-structured qualitative interviews were completed with a convenience sample of *s*tudy participants with HIV and OUD (*n* = 37) randomized to XR-NTX in five HIV clinics between 2018 and 2019. All participants approached agreed to be interviewed. Interviews were digitally recorded, professionally transcribed, and analyzed using thematic analysis.

**Results:**

Participants included women (43%), African Americans (62%) and Hispanics (16%), between 27 to 69 years of age. Individuals who completed XR-NTX induction *(n* = 20) reported experiencing (1) readiness for change, (2) a supportive environment during withdrawal including comfort medications, and (3) caring interactions with staff. Four contrasting themes emerged among participants (*n* = 17) who did not complete induction: (1) concern and anxiety about withdrawal including past negative experiences, (2) ambivalence about or reluctance to stop opioids, (3) concerns about XR-NTX effects, and (4) preferences for other medications.

**Conclusions:**

The results highlight opportunities to improve initiation of XR-NTX in high-need groups. Addressing expectations regarding induction may enhance XR-NTX initiation rates.

*Trial Registration* ClinicalTrials.gov: NCT03275350. Registered September 7, 2017. https://clinicaltrials.gov/ct2/show/NCT03275350?term=extended+release+naltrexone&cond=Opioid+Use.

## Background

Approximately 2 million individuals in the U.S. have an opioid use disorder (OUD) [1] and only a fraction receive medications for opioid use disorder (MOUD) treatment [2, 3]. Untreated OUD is associated with increased HIV risk behaviors [4, 5], decreased receipt of antiretroviral therapy (ART) [5–7], decreased ART adherence [5, 8–10], and decreased HIV viral suppression [11, 12]. Treatment of OUD can increase engagement in HIV care and enhance health outcomes [11, 13]. MOUD with an opioid receptor agonist (methadone), partial agonist (buprenorphine), or opioid antagonist (extended-release naltrexone) effects at the opioid mu receptor facilitate recovery from OUDs [14]. Only 36% of specialty substance use disorder treatment organizations in the U.S., however, provide MOUD [15].

Despite compelling evidence that MOUD is effective [15–19], these medications remain underutilized [20]. This is due in part to the need for daily dosing. Recent advances are changing the landscape; some partial and full antagonist treatments such as extended-release formulations of naltrexone (XR-NTX) and buprenorphine provide alternatives to the daily dosing requirements of methadone. XR-NTX, a deep muscle injection that lasts 28 days, eliminates the need for daily dosing. While long-acting formulations may improve treatment adherence, a recent study found that it was more difficult to initiate XR-NTX than buprenorphine in patients with OUD [19]; the process of initiating XR-NTX so that someone receives their first injection (a.k.a. “induction”) requires an opioid-free state several days prior to initiation, and opioid withdrawal can be difficult to complete even in inpatient settings with supportive medication [21]. In contrast, buprenorphine induction does not require complete abstinence from opioids; first dose of buprenorphine may be administered 12–24 h after last use of opioids. Multi-site trials in North America and Europe noted that some individuals randomized to XR-NTX did not complete induction; induction rates were enhanced in inpatient settings (75%–90%) compared to primarily outpatient settings (68%) [19, 22–24].

A 51-patient pilot study demonstrated the feasibility of XR-NTX for the treatment of opioid and alcohol use disorders in two HIV primary care clinics [22]. Mean days of opioid use in the past 30 days decreased in both the treatment as usual group (i.e., methadone or buprenorphine) (17.3 to 4.1 days) and the XR-NTX group (20.3 to 7.7 days). HIV suppression increased from 67 to 80% for XR-NTX and 58% to 75% for treatment as usual [22]. However, only 42% of participants with OUD assigned to XR-NTX completed induction [22]. Based on these pilot data, the “Comparing Treatments for HIV-Infected Opioid Users in an Integrated Care Effectiveness Study (CHOICES) Scale-up study” (CTN-0067) was redesigned and expanded to five HIV clinics willing to randomize patients to either opioid agonist therapy or opioid antagonist therapy. Of 55 participants randomized to XR-NTX, 26 completed an XR-NTX induction.

### Methods

We contacted 37 of the 55 participants randomized to XR-NTX to conduct semi-structured, in-depth interviews (20 induced, 17 not induced) from five participating HIV clinics between 2018 and 2019, during early study implementation. Study participants were selected because they completed or did not complete a first XR-NTX injection, and the interview guides and analysis were designed to assess how the experiences differed. The semi-structured research guides were created by the core qualitative research team. We used a convenience sampling approach (based on participant and clinic scheduling availability) and all participants approached for the interview agreed to participate. Qualitative interviews assessed the opioid withdrawal and XR-NTX induction experience for participants randomized to XR-NTX to identify more effective strategies for initiating opioid antagonist therapy. For those who did not initiate XR-NTX, study participants were interviewed about their induction attempt experience. Interviews were digitally recorded, professionally transcribed, and examined using thematic analysis [25].

### Participant eligibility

Individuals with moderate or severe OUD and an HIV viral RNA level of ≥ 200 copies/ml were eligible for the CHOICES Scale Up Study. Participants randomized to XR-NTX were eligible for qualitative interviews and recruited during their scheduled clinic appointments for MOUD, HIV care, or study visits. Potential interview participants were informed of the overall aims of the qualitative study and invited to participate in either face-to-face or telephone interviews. All individuals invited to complete interviews agreed to participate. Participants received a $50 gift card for their participation. The study’s Central IRB reviewed the interview guides and approved an information sheet for study participants [26].

### Interviews

The semi-structured research guide was created by the core qualitative research team; core research questions were asked but the interview allowed for follow-up probes by the investigator based on answers to the original question. Two highly experienced qualitative interviewers (KH and RB), who were not responsible for enrolling participants in the clinical trial, conducted the interviews. Face-to-face interviews (n = 19) were conducted in a private clinic room with only the interviewer and the participant present. In the case of telephone interviews (n = 18), study staff facilitated the initiation of the call and then left the room so that the conversation between the interviewer and respondent was private. Interviews lasted between 20 and 40 min, averaged 33 min, and were digitally recorded. Interview topics included: (1) participant characteristics, (2) current and prior HIV care, (3) history of alcohol and drug use, (4) substance use treatment history, (5) social supports, and (6) views on medications for OUD including withdrawal and induction experiences. Interviews were completed, recorded and professionally transcribed between July 2018 and November 2019.

### Analysis

Given the research aim to elicit how respondents constructed their own lived experience, a Constructivist paradigm [27] was used. Within that paradigm, the team followed Braun and Clarke’s [25] Thematic Analysis (TA) as a specific approach to analysis. When applying TA, the six-step procedures defined by Braun and Clarke were applied. Themes were identified which related to opioid withdrawal and XR-NTX induction experiences and barriers to induction using an inductive approach at the semantic level. Three PhD level investigators (KH, RB, DM) with extensive experience in qualitative data collection, analysis and publication [21, 28–31] drafted a list of preliminary themes after reading interview transcripts and two coded transcript themes (KH, RB). An inter-coder reliability process assessed coding discrepancies and a third coder (DM) adjudicated the coding [32]. Ten percent of the transcripts were double-coded to achieve an inter-coder reliability rate of 85%. After achieving agreement on the themes, representative quotations were selected by consensus. Atlas.ti 8 software facilitated data processing and quotation retrieval.

## Results

Participants included women (43%), African Americans (62%) and Hispanics (16%), between 27 to 69 years of age (see Table 1). Twenty participants who had completed XR-NTX induction reported (1) readiness for change, (2) a supportive environment during withdrawal including comfort medications, and (3) caring interactions with staff. Four contrasting themes emerged among participants (*n* = 17) who did not complete induction: (1) concern and anxiety about withdrawal including past negative experiences, (2) ambivalence about stopping opioids, (3) concerns about XR-NTX effects, and (4) preferences for other medications.

**Table 1:** Participant characteristics

N = 37		
Age	n	%
Mean Age	51 years old	
27–39	7	19
40–59	23	62
60–69	7	19
Gender		
Men	21	57
Women	16	43
Race		
White	8	22
African American or Black	23	62
Hispanic	6	16
Education		
Less than High School graduate	18	48
High School graduate or GED	8	22
Some college	8	22
Associates degree	1	3
Bachelor’s degree	2	5
Marital status		
Married/life partner	2	5
Divorced	8	22
Never married	21	57
Separated	4	11
Widowed	2	5
Employment status		
Employed	4	11
Unemployed or looking for work	19	51
Disabled	10	27
Retired	4	11

### Themes associated with successful XR-NTX induction

#### Readiness for change

For respondents who successfully completed induction, the most common theme which emerged was around their readiness to undergo induction. Before persons with OUD begin treatment with XR-NTX, they must be opioid-free prior to the first injection. If opioids are present, the XR-NTX will displace the opioids from their receptors and produce symptoms of opioid withdrawal, a constellation of uncomfortable, often severe, flu-like symptoms. All CHOICES study participants reported common opioid withdrawal symptoms such as pain, diarrhea, nausea, headaches, and backaches. Given the discomfort of opioid withdrawal, participants were asked to talk about their motivation for induction onto XR-NTX. Participants spoke of their families, a desire to improve their lives, and being tired of life on opioids:

*I think the will. Yes. Yes. … My wife, my children. I have grown-up kids and I don’t want them to continue seeing me like this. … My wife has always stayed with me… She has always been there with me.[Case ID 32]*

*I just told myself ‘I wanted a better life’. [Case ID 19]*

*I'm really actually– I'm tired of getting– I'm tired… I tell you I am tired. [Case ID 27]*

Another related how their experience with overdose and fear of dying provided motivation for induction onto XR-NTX:

*I OD'ed. Yeah. And I never overdosed on any substance before and every day I had to build up my mind, my body and my spirit to not use. It was a hard thing to do. It was a struggle; my body is craving something and me saying no because I could die. It was very hard but I did it. [Case ID 24]*

One respondent recognized that study incentives helped him complete the painful process of withdrawal and gave him a sense that participation in the study was beneficial.

*The study was a way to get extra money and based on the information they gave me about the risk and what they were trying to accomplish I felt like I could do it and … it was a worthwhile endeavor for me. [Case ID 25]*

Similar themes related to motivation to change were echoed in advice that participants had for others who were considering XR-NTX. Participants stressed the importance of readiness and individual commitment to stop opioid use and the importance of wanting to stop opioid use.

*Just make sure that you really want to stop using because it do work. You have to be ready to stop using and so be ready. Some be ready and they be scared but it works. They don't need to be scared but just give yourself a chance. [Case ID 18]*

*I think other people who want to get clean, it's going to work perfectly. For other people who are doing it for the wrong reasons, I think it's going to keep them from getting high but it's not going to stop them from wanting to use. The will has to be there. The body will follow. [Case ID 30]*

### A supportive environment during withdrawal including comfort medications

Another commonly discussed theme included discussion about the environment in which the respondents underwent withdrawal. Participants completed opioid withdrawal in inpatient facilities for medically managed withdrawal, jails, at home, and on the streets with varying levels of comfort and success. Many participants had difficulty accessing inpatient medically supervised withdrawal due to long waiting lists. While some participants withdrew from opioids successfully at home, others reported that home was a suboptimal location due to ongoing availability of opioids or concerns about safety.

*[If I tried to] withdraw at home, I would end up using. I made up my mind to go on the treatment because I really wanted the injection [XR-NTX]. [Case ID 33]*

*Prior to getting the shot [XR-NTX], I tried to detox because I didn't want to go into [inpatient] detox. I lasted about maybe a week or two, and that was on the streets. That was hell, because everywhere around me, everyone was using. [Case ID 30]*

A respondent who completed withdrawal at home mentioned that their friends in recovery checked on them regularly throughout the process. They observed that they would have been more comfortable in an inpatient facility if that had been an option. Although their brothers and sisters checked on them regularly, for example, this participant would have preferred admission to an inpatient facility for withdrawal management.

*You need to be like somewhere where they can monitor your vitals. You can't do it by yourself. I had my apartment. They were there with me for the seventy-two hours but you need someone in case your body just stops and you can't do it alone. All my brothers and sisters are in recovery now and they were calling and coming back. [Case ID 24]*

Participants who used medically managed withdrawal facilities attributed their success to access to ancillary medications and a supportive environment.

*You know, you can relax and rest. You get the rest you need and just focus on you. And then you have medical assistance with problems or struggles or anything like that or if you feel like crap you don't go, you know, and look to use. You just, you know, get the help that you need to get through the medical stuff. [Case ID 20]*

### Caring interactions with others

Though less commonly cited, a final theme among the inducted participants was the critical role of supportive study staff in helping them complete opioid withdrawal and XR-NTX induction. Participants commented that study staff provided both educational, operational, and emotional support. These supports included information, flexibility to accommodate life responsibilities such as child care, and medications that helped to alleviate withdrawal symptoms.

*Well, yes, yes, they did treat me well here, truly, they treated me like I was family. I would come here with my daughter because my wife couldn’t look after her. So, sometimes, I had to take care of my daughter. Here, they took care of her while I was being seen and all, they have treated me very well here. [Case ID 32]*

*They did everything–awesome. Very awesome and very, you know, good at explaining everything and good at getting through all the paperwork. [Case ID 26]*

*My doctor—my psychiatrist gave me the pills for when I get sick, like stomach cramps, runny nose, something for my, you know, the bones when I be aching and stuff like that, she gave me medication for all that.* [Case ID 02]

Emotional support included compassion, encouragement, and helping participants cope with fears and anxieties.

*I don't like needles at all so I had to mentally get ready for that. So when Dr. (name) was there to do the shot, okay, the lady (nurse) was like I am going to hold your hand like this. Okay, ready. Go. And he just like, was already done. Already put it in and took it out and I'm still holding her hand waiting. I didn't even feel anything.” [Case ID 26]*

*My caseworker and [study staff helped] because they talked with me and I wanted to prove to them that I could do right. They let me know that there is somebody in this world that wants to see you get right. And this … made me realize that some people in this world will help you. [Case ID 19]*

### Themes associated with unsuccessful XR-NTX induction

Study participants who were unable to complete induction emphasized fear of withdrawal, ambivalence about stopping drug use, misperceptions of XR-NTX and a preference for opioid agonist therapy.

### Concern and anxiety about withdrawal including past negative experiences

The most commonly discussed theme which emerged concerned worry about withdrawal. Among individuals randomized to XR-NTX, fear of completing opioid withdrawal was a common barrier to induction. They described the physical and emotional symptoms associated with previous opioid withdrawal, including generalized pain, nausea, vomiting, diaphoresis and severe anxiety. A 27-year-old woman reported:

*I was really excited to get it and everything. That's what I wanted, was the [XR-NTX], but every time I make a plan to go into detox, right there I am ready and everything and then I can't do it and back out. They come and pick me up and I'm in the car and…I have really, really bad anxiety. So it's tense… It's just the fear of everything. [Case ID 11]*

A middle-aged respondent experiencing homelessness expressed that his inability to stop all opioids for more than a day had to do with being homeless and lacking a structured environment. He reported attempting to withdraw on his own to prepare for induction: *“I tried to detox but it wouldn't happen for me.” [Case ID 16].* He was unaware of any medically supervised withdrawal centers in the area.

Some participants believed that transitioning directly from heroin or fentanyl was extremely difficult or impossible. A 40-year-old man explained that he attempted going through withdrawal for the XR-NTX injection “*three or four times*” at home. He contemplated:

*I know people take the shot but how do they get clean by taking it, you know what I mean? I think you all should have a way for them to methadone down, then detox off methadone, then probably take the shot. But that straight coming from heroin to the shot, that's the hard stuff. Cold turkey. That's why some people still out there now. They tried it, it don't work. [Case ID 06]*

He had been injecting heroin for 22 years and reported “*buying methadone off the street”* to taper off of heroin in preparation for his XR-NTX induction, but ended up enrolling in a methadone program instead:

*[The study staff said] All right but you got to be totally clean, you can't take any methadone or have none of that stuff in your system, nothing. It's got to be like cold. And especially injecting, my withdrawals are more extreme than I think the ones who snorted. So I tried it. I tried it about two or three times but you can't take nothing with opioids in it. Nothing to calm down your pain. It wasn't working at all. Wasn't working at all. So I just started methadone. So it's been hard but now I'm on the methadone program and I ain't used in about a month and a half so it's been good. [Case ID 06]*

A female respondent reported she “*had once tried to commit suicide because I wanted to stop using so bad.”* [Case ID 17] After 7 days of medically supervised withdrawal, however, a naloxone challenge precipitated withdrawal.

*I want the cravings to go away and that's what the [XR-NTX] shot should do. I went through detox for seven days when I was doing the study, trying to get on the … shot but fentanyl was in my system before I went in the detox and even after seven days, after finishing the detox, fentantyl was still in my system so I wasn't able to get the shot. They gave me the [naloxone] to try to take it out of there but it instantly made me sick and I didn't want to do another dose of [naloxone] because it made me so sick and then I went out and got me a bag of heroin that day. It made me go through withdrawals really bad, really quick. I was ready to leave instantly after that.* [Case ID 17]

As a result, she began a methadone program and, at the time of the interview, was still using some heroin to help with cravings. Her ultimate goal was to complete withdrawal from opioids so that she can begin treatment with XR-NTX:

*I have an appointment with my counselor next Wednesday to talk about me starting to decrease from the methadone so that I can get down so they can switch me to the [buprenorphine] again and then she can switch me to the [XR-NTX] shot.* [Case ID 17]

### Ambivalence about or reluctance to stop opioids

Ambivalence about stopping opioid use was common among participants and some identified the positive effects of opioid use that they would lose on XR-NTX. The potential negative effects of XR-NTX were also of concern. Participants feared, for example, that either they would have pain and be unable to manage it without opioids, or they felt that opioids helped them cope with difficult life experiences. Others were simply not ready to stop. Two participants summarized their feelings:

*I don't want to stop yet and get this twenty day blocker. It's definitely great for somebody who really wants to stop and doesn't have reservations. [Case ID 10]*

*I'm not ready to get off heroin. I'm being honest. [Case ID 09]*

A respondent felt that opioids helped him maintain employment by keeping him “*well*”. He was providing financial support to his brother and therefore prioritized his work over treatment:

*Like when I am working, I just keep myself well….Some of my family tells me you know, it's better that you just go into treatment for a while, get yourself together and then things will probably fall into place and get better. Which is true but by me just losing my mom a few months ago mentally I tell myself I need to just work so I can help my brother. [Case ID 03]* One respondent discussed the complexities of avoiding withdrawal and how that related to his feelings about treatment with XR-NTX:

*For me, because me being used to getting high for so long over twenty-seven years, it's going to be hard… I think I need to be on some medication that would get me close to that standard than to just do cold turkey. That's still cold turkey to me—when you don't get high period, when you are used to getting high. So it's still a problem and I don't want it to be a problem. Mentally.” [Case ID 15]*

*Similarly, a 55 year old man talked about continuing to use heroin: “Cause I been doing it a long time I probably would have had to do some more even if I did take the shot.” [Case ID 16] When asked if he would prefer to continue to use heroin, he divulged, “For the moment, I am still using but I am really thinking about trying to get off….I thought I felt ready but I guess I am not, yet. [Case ID 16].*

A 30-year-old respondent who did manual labor for a living described how the positive effects of using heroin, such as pain relief, outweighed the negative experiences like withdrawal and other possible consequences.

*No, [I am not ready to stop]. … I don't know, it's weird, I want to but I don't. When I stop, like I have physical problems and when I'm high I don't have no aches, no pains, just when I'm like not actually high, just living that lifestyle, I don't have any pain unless I just wake up in the morning and I am just feeling a little sick. That's the only thing but when I get clean I have aches and pains and that's-- it sucks. … I don't know, I just don't know, I got to see. I have been dodging bullets I'd say but you know, I just need to bite the bullet and go see doctors and psychiatrists you know, but…[Case ID 10]*

### Concerns about XR-NTX effects

Some patients expressed negative perceptions about XR-NTX including fear of experiencing opioid withdrawal, concerns about unmanaged pain, and the fact that it is not an opioid agonist. In addition, participants had previously received negative or potentially confusing information about the extended-release formulation and were concerned that they would still have cravings, try to use higher amounts of opioids and increase the risk of overdose, or potentially increase use of stimulants when they were unable to use opioids. When asked about his perception of XR-NTX, a 36 year old Hispanic male reported:

*Maybe it has to do with that it was females that were giving the [negative] feedback, just the females acted that way so, anyway, the females that told me about it, one of them told me that she was still sick after taking a shot of [XR-NTX]. The other one told me she turned into a crack addict-- a crack monster. [Case ID 10]*

When asked what she had heard about XR-NTX, a female respondent reported that she had heard it was “*a good thing but don't you never do the dope because it's going to kill you. Be ready you know in your heart you won't do it no more*”. *[Case ID 01].*

### Preferences for other medications

Though less commonly cited, a few participants assigned to XR-NTX preferred opioid agonist therapy with buprenorphine or methadone, particularly when they had prior beneficial experiences with either medication. In some cases, participants were exposed to buprenorphine or methadone during medically supervised withdrawal admissions and opted to remain on those medications even though they had been randomized to XR-NTX.

A male participant reported that after study staff found him an inpatient medically supervised withdrawal center, he was still testing positive for opioids. He was subsequently moved to a hospital, where he was provided buprenorphine.

*So, the next step from the detox was the inpatient treatment at [name] … and I was there for four months … Did good while I was there. I stayed clean. I was introduced to the [buprenorphine] and it was working for me. I'm still using it. It's still working for me. [Case ID 12]*

In collaboration with his HIV provider, he decided it was best for him to remain on buprenorphine rather than initiate XR-NTX. Another individual reported a similar scenario; after being introduced to buprenorphine during his medically supervised withdrawal, he mulled over the decision about whether to switch to XR-NTX:

*Well, when I heard, you know, at first I was going to switch over and then something just said, no, no, no. Stay with what you are doing because it's working. So, I stayed on the [buprenorphine]. I was satisfied. [Case ID 13]*

## Discussion

The current study suggests that individuals who completed XR-NTX induction reported (1) readiness for change, (2) a supportive environment during withdrawal including comfort medications, and (3) caring interactions with staff. Four contrasting themes emerged among participants (*n* = 17) who did not complete induction: (1) concern and anxiety about withdrawal including past negative experiences, (2) ambivalence about or reluctance to stop opioids, (3) concerns about XR-NTX effects, and (4) preferences for other medications. Of note, participants who experienced inpatient medically managed withdrawal seemed to have an advantage over those who did not have access. Inpatient treatment served the purpose of avoiding hazards associated with withdrawal and return to use; in this environment, supportive medications can make the early hours and days of withdrawal as comfortable as possible while removing the patient from their use environment. This is consistent with other studies showing that linking patients from inpatient managed withdrawal programs to long‐term long term MOUD treatment reduces illicit opioid use and increases days of treatment [33, 34]. To our knowledge, this is the first qualitative report of patients experiences with successful, and unsuccessful XR-NTX induction and advances understanding of how to better support patients in initiation of XR-NTX. The paper’s contribution to the literature is that it documents study participants’ experiences who tried but were unable to withdraw from opioids and did not receive an injection of extended-release naltrexone. Patients who completed induction also reported difficulty withdrawing but had sufficient social and environmental support to overcome withdrawal symptoms and receive the first injection of XR-NTX.

The theme of motivation, or “readiness to change”, also played an important role and is a well-known predictor of treatment engagement [35]. Clinician assessment of a patient’s motivation and use of techniques to advance progression along stages of readiness such as Motivational Interviewing may be helpful.

Patient preference also influenced participant enthusiasm for treatment initiation, with some participants indicating that their preference for opioid agonist treatment dampened their enthusiasm for XR-NTX induction. Patient preference was similarly associated with treatment outcome in a large comparative effectiveness trial of XR-NTX versus buprenorphine [19]. National treatment guidelines endorse shared decision-making in choosing medications for OUD [36]. Development of patient-facing decision aids, as developed for other medical treatments, may enhance patient-centered, informed decision-making in choice of OUD treatment [37]. Our results are also consistent with another recent investigation which found that it is more difficult to induct patients onto XR-NTX than buprenorphine, though requirements for a negative-buprenorphine urine sample before XR-NTX induction may have exacerbated the underlying withdrawal tolerance issues [19]. Additionally, in a previous study of CHOICES study staff, Hoffman et al. [21] found that a specific medication-related barrier to induction was patient fear of opioid abstinence required prior to XR-NTX induction. Similar results have been found in other studies of individuals using stimulants and opioids [31]. Overcoming these barriers to XR-NTX induction may require additional counseling on how XR-NTX works, expectations for induction, aggressive advance treatment of opioid withdrawal symptoms (e.g., standing doses of clonidine, trazodone for sleep, hydroxyzine for anxiety, etc.), and respect for patient preferences for opioid agonist versus antagonist treatment. Study participants perceived that withdrawal from fentanyl was more difficult than other opioids and a subsequent quantitative analysis confirmed that induction rates were substantially lower among individuals with fentanyl positive urine screens (adjusted hazard ratio = 0.09, 95% confidence interval 0.03 to 0.24) [38]. This study points to the need for new clinical interventions to manage fentanyl withdrawal.

Some limitations of the study should be acknowledged. It is important to note that this study was exploratory and assessed barriers and facilitators to XR-NTX induction among a convenience sample of individuals already enrolled in a treatment trial. It did not include all patients in the trial, and thus may not represent all views. Another limitation concerns the generalizability of the findings. The relatively small sample of individuals enrolled in a trial and receiving compensation may not reflect the general population of those seeking treatment for opioid use disorder. Additionally, there was heterogeneity of sample demographics, interview method (in-person, telephone), and induction settings.

## Conclusions

The results highlight areas that need to be addressed in order to improve uptake of XR-NTX. In the U.S., new policies and guidelines are increasingly proposed and adopted to address the opioid epidemic but more is needed to reach high-needs populations, such as those living with uncontrolled HIV disease [20]. Successful induction onto XR-NTX can be associated with a supportive and safe setting for withdrawal management, use of ancillary medication to minimize opioid withdrawal symptoms, and support from staff, family and friends. Shared decision making that prioritizes patient preferences helps patients better understand the effects of opioid agonist and opioid antagonist therapies may improve initiation of medications for opioid use disorder treatment.

## Data Availability

CTN quantitative data are released to the public one year after the publication of primary outcome results. See CTN data share site: https://datashare.nida.nih.gov/index.php/data?field_clintri_study_division_target_id=2&field_clintri_keywords_target_id=All. Qualitative data may be obtained by contacting the corresponding author.

## Abbreviations

XR-NTX: Extended-Release Naltrexone
SUD: Substance use disorder
OUD: Opioid use disorder
